# Supplementary material for: Osteonecrosis in Korean Paediatric and Young Adults with Acute Lymphoblastic Leukaemia or Lymphoblastic Lymphoma: A Nationwide Epidemiological Study
Source: J Clin Med. 2022 Apr 28;11(9):2489. doi: 10.3390/jcm11092489 (PMC9105090; doi:10.3390/jcm11092489)
Supplement: Supplementary file 1 [file jcm-11-02489-s001.zip › jcm-1656370-supplementary.pdf]

Supplementary Materials

Table S1. Risk factors of avascular necrosis (young adult group).

Risk Factors		ALL			Young Adult Group (n=1517)		
		**No AVN (n=4703)**	**AVN (n=158)**	***p*-value ***	**No AVN (n=1452)**	**AVN (n=65)**	***p*-value ***
		Median (Q1, Q3) or n (%)	Median (Q1, Q3) or n (%)		Median (Q1, Q3) or n (%)	Median (Q1, Q3) or n (%)	
Age		12 (4, 23)	17 (13, 26)	<0.001	29 (20, 39)	28 (20, 39)	0.121
Sex	Male	2800 (97.05)	85 (2.95)	0.149	873 (95.72)	39 (4.28)	0.984
Radiotherapy	Yes	944 (94.87)	51 (5.13)	<0.001	497 (95.39)	24 (4.61)	0.655
HSCT	Yes	1329 (95.41)	64 (4.59)	<0.001	792 (95.08)	41 (4.92)	0.176
Steroid use**	Dexamethasone	1074 (96.67)	37 (3.33)	0.141	543 (95.77)	24 (4.23)	0.399
	Prednisone	1953 (96.68)	67 (3.32)		334 (94.35)	20 (5.65)	
	Dexamethasone + Prednisone	1144 (96.22)	45 (3.78)		392 (96.08)	16 (3.92)	
	Not used	532 (98.34)	9 (1.66)		183 (97.34)	5 (2.66)	
Asparaginase use	Yes	3205 (96.59)	113 (3.41)	0.371	583 (94.80)	32 (5.20)	0.145
Anthracycline use **	Yes	3020 (95.90)	129 (4.10)	<0.001	1153 (95.37)	56 (4.63)	0.190

*Wilcoxon rank sum test; HSCT, hematopoietic stem cell transplantation; **during 1st 60 days after diagnosis

Table S2. Univariate and multivariate analysis associated with AVN (young adult group).

**Risk Factors**			**Univariate**		**Multivariate _ON**		**Multivariate_Death**	
	**level**	**Median (Q1, Q3) or n (%)**	**HR (95% CI)**	***p*-value ***	**HR (95% CI)**	***p*-value ***	**HR (95% CI)**	***p*-value ***
Age		29 (20.39)	0.970 (0.930, 1.012)	0.162	0.972 (0.931, 1.015)	0.196	1.018 (1.003, 1.033)	0.018
Sex	Male	912 (60.12)	1.068 (0.650, 1.755)	0.795	1.000 (0.606, 1.651)	0.999	1.086 (0.911, 1.295)	0.359
Radiotherapy	Yes	521 (34.34)	1.290 (0.772, 2.154)	0.331	1.019 (0.575, 1.804)	0.949	1.239 (1.012, 1.517)	0.038
HSCT	Yes	833 (54.91)	1.638 (0.963, 2.787)	0.069	1.324 (0.718, 2.442)	0.369	1.193 (0.965, 1.474)	0.103
Steroid	Dexameth	567	2.664 (1.011,	0.047	1.846	0.3	2.113 (1.407,	<0.001

use**	asone	(37.38)	7.017)		(0.564, 6.045)	11	4.174)	
	Prednisone	354 (23.34)	2.810 (1.051, 7.508)	0.039	1.687 (0.506, 5.618)	0.395	1.277 (0.831, 1.962)	0.266
	Dexamethasone + Prednisone	408 (26.90)	2.647 (0.965, 7.263)	0.059	1.620 (0.470, 5.578)	0.445	1.685 (1.099, 2.584)	0.017
	Not used	188 (12.39)	1 (ref)		1 (ref)			
Asparaginase use	Yes	615 (40.54)	1.783 (1.094, 2.908)	0.020	1.510 (0.862, 2.644)	0.150	1.964 (1.622, 2.377)	<0.001
Anthracycline Use**	Yes	308 (20.30)	2.316 (1.141, 4.698)	0.020	1.287 (0.514, 3.219)	0.590	1.056 (0.784, 1.423)	0.720

* Wilcoxon rank sum test; HSCT, hematopoietic stem cell transplantation; ** during 1st 60 days after diagnosis.
